# ALKBH5 prevents hepatocellular carcinoma progression by post-transcriptional inhibition of PAQR4 in an m6A dependent manner

**DOI:** 10.1186/s40164-022-00370-2

**Published:** 2023-01-06

**Authors:** Weijian Wang, Qibo Huang, Zhibin Liao, Hongwei Zhang, Yachong Liu, Furong Liu, Xiaoping Chen, Bixiang Zhang, Yan Chen, Peng Zhu

**Affiliations:** 1 Hepatic Surgery Center, Tongji Hospital, Tongji Medical College, Huazhong University of Science and Technology, Wuhan, 430030 China; 2 Hubei Key Laboratory of Hepato-Pancreato-Biliary Diseases, Wuhan, 430030 China; 3 Department of General Surgery, Tongji Hospital, Tongji Medical College, Huazhong University of Science and Technology, Wuhan, 430030 China

**Keywords:** ALKBH5, Methylation, PAQR4, AKT, HCC

## Abstract

**Background:**

N6-methyladenosine (m6A) is a prevalent modification of mRNA and is known to play important roles in tumorigenesis in many types of cancer. The function of N6-methyladenosine (m6A) RNA methylation depends on a variety of methyltransferases and demethylases. AlkB homolog 5 (ALKBH5) is a demethylase, and its biological function has not been completely explored in HCC.

**Results:**

ALKBH5 is downregulated and has antitumor effects in HCC cells. In addition, Progestin and AdipoQ Receptor 4 (PAQR4) was identified as a downstream target of ALKBH5 based on transcriptome sequencing and validation studies. We found that ALKBH5 decreases PAQR4 mRNA and protein expression in an N6-methyladenosine (m6A)-dependent manner. The study also showed that ALKBH5 changes PAQR4 expression via the m6A reader IGF2BP1. In both in vivo and in vitro experiments, PAQR4 showed a strong association with the development of HCC. Finally, we found that PAQR4 interacts with AKT and enhances PI3K/AKT pathway activation.

**Conclusions:**

ALKBH5 inhibits HCC growth by downregulating PAQR4 expression in an m6A-dependent manner, therefore suppressing PI3K/AKT pathway activation.

**Supplementary Information:**

The online version contains supplementary material available at 10.1186/s40164-022-00370-2.

## Background

Hepatocellular carcinoma (HCC) is still one of the most prevalent malignancies and is the fifth leading cause of cancer death worldwide [1, 2]. Despite achievements in the prevention of HCC, the incidence rate of HCC worldwide is still rising, while the incidence rate of HCC has been declining in some areas in recent years [3–5]. To date, some patients have benefited from current advances in standardized treatment. However, the long-term survival rate of patients with HCC is low [6], which is attributed to a weak response to chemotherapeutic agents and to metastasis and recurrence of hepatic cancers after resection [7, 8]. The abovementioned situations have inevitably led to treatment failure in HCC. Therefore, we need to explore the specific mechanisms involved in the recurrence and development of HCC.

As a unique RNA modification, m6A modification was first identified in the 1970s. However, it was after the discovery of FTO as the first RNA demethylase in 2011 [9] that the methylation of RNA was regarded as a reversible process. Since then, research on m6A RNA modification has arisen to fill the gap in this field. Dynamic regulation of RNA methylation mainly depends on ‘‘writers’’ and ‘‘erasers’’, which function as dedicated RNA methyltransferases and demethylases, respectively [10]. In the past few years, with the development of high-throughput sequencing technology and new anti-m6A antibodies, the role of m6A modification in hepatic cancers has been increasingly discussed [11].

In HCC, the m6A methyltransferase methyltransferase-like 3 (METTL3) contributes to HCC progression by methylation of SOCS2 and Snail [12, 13]. Similarly, the methyltransferase methyltransferase-like 14 (METTL14) suppresses metastasis in HCC by modulating the methylation levels of microRNA 126 and USP48 [7, 14]. However, the potential roles of the demethylases fat-mass and obesity-associated protein (FTO) and AlkB homolog 5 (ALKBH5) in HCC have remained unexamined. There have been few studies of FTO in liver cancer, while the results of studies of ALKBH5 in HCC are inconclusive [15].

ALKBH5 was first reported as a mammalian demethylase in 2013 [16]. Soon after that, many studies were conducted on ALKBH5. ALKBH5 maintains the tumorigenicity of glioblastoma stem-like cells (GSCs) through the regulation of FOXM1 in a methylation-dependent manner [17]. In intrahepatic cholangiocarcinoma (ICC), ALKBH5 regulates the tumor immune microenvironment and immunotherapeutic efficacy via modulating the methylation of PD-L1 mRNA [18–20]. In ovarian cancer, ALKBH5 promotes cisplatin resistance in cancer cells by modifying its m6A target gene JAK2 to activate the JAK2/STAT3 signaling pathway[21]. These findings demonstrate that ALKBH5 is involved in carcinogenesis, tumor formation and the immune microenvironment in many types of cancer. In HCC, the malignancy of hepatocellular carcinoma cells is suppressed by ALKBH5 via m6A-guided suppression of the oncogenic driver LYPD1 [22]. However, another article reported that ALKBH5 promotes hepatocellular carcinogenesis in HBV-related HCC and catalyzes m6A demethylation of HBx mRNA to sustain its expression [23]. Thus, the role of ALKBH5 in HCC remains to be clarified.

Here, we found that ALKBH5 expression was decreased in HCC tissues. We discovered that ALKBH5 suppressed HCC cell proliferation and invasion in vitro. By transcriptome sequencing and subsequent validation through filtering, we found that PAQR4 mRNA was methylated and modified by ALKBH5, which influenced the stability of PAQR4 mRNA in an IGF2BP1-dependent manner. Additionally, we suggested that PAQR4 can promote the proliferation, migration, and invasion of HCC cells in vivo and in vitro. Furthermore, we found that PAQR4 promoted the malignant behavior of HCC cells through the PI3K/AKT signaling pathway. Overall, we found that PAQR4, a target gene of ALKBH5 for demethylation, promotes HCC cell proliferation, migration, and invasion via the PI3K/AKT signaling pathway.

## Results

### The ALKBH5 protein level is decreased in HCC and suppresses HCC proliferation and invasion

**Fig. 1:**
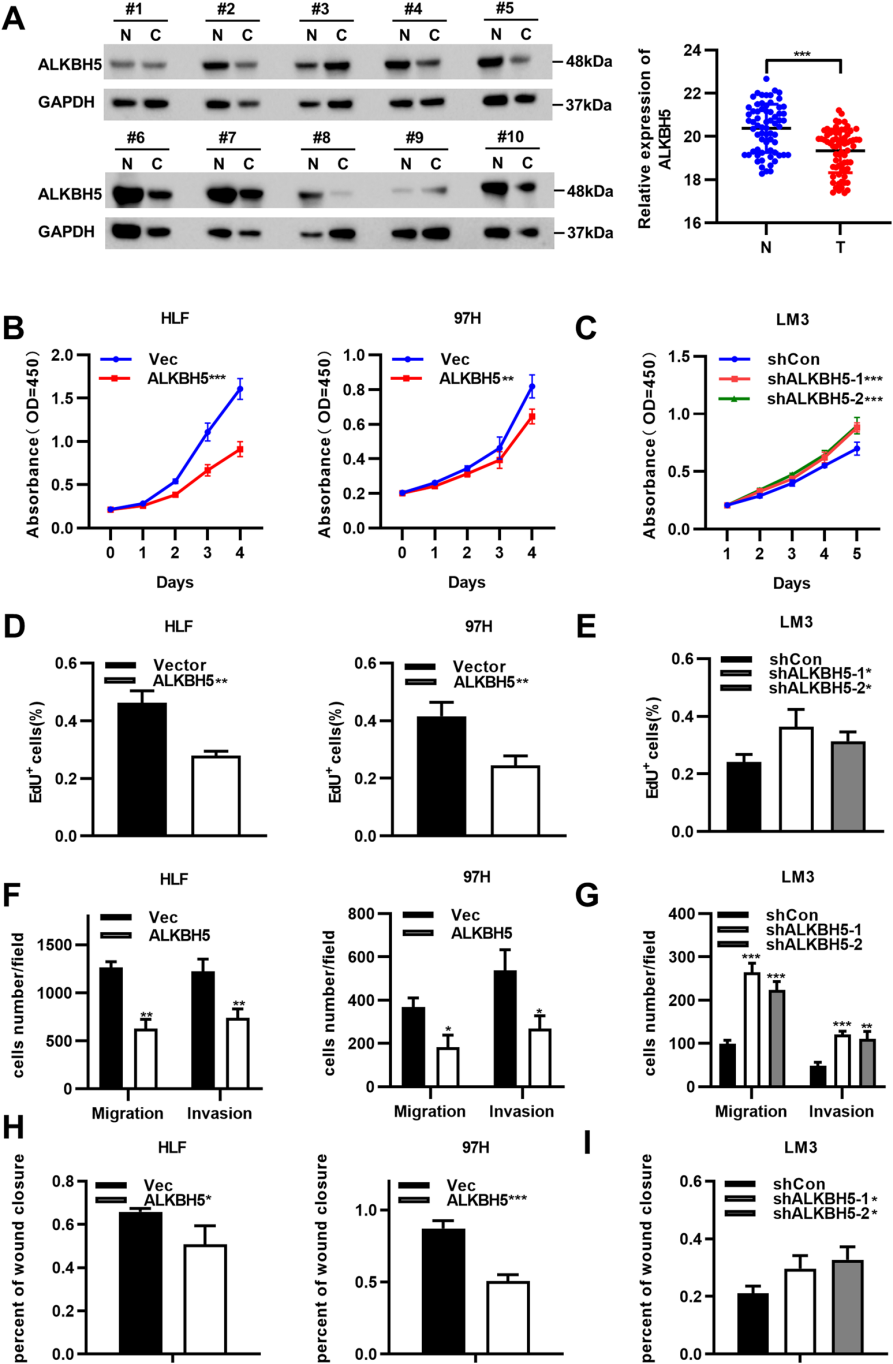
ALKBH5 is decreased in HCC and suppress HCC proliferation and invasion in vitro. **A** Protein level of ALKBH5 was detected by Western bolt assay in paired HCC samples (n = 60). CCK8 and EdU assays confirmed that overexpression of ALKBH5 inhibit proliferation of 97H and HLF (**B**, **D** and Additional file 1A), whereas knockdown of ALKBH5 promoted cell proliferation in LM3 (**C**, **E** and Additional file 1A). Transwell and wound healing assays showed that overexpression of ALKBH5 suppress the capability of migration and invasion in 97H and HLF (**F**, **H** and Additional file 1B, C). In contract, knockdown of ALKBH5 promoted migration and invasion in LM3 (**G**, **I** and Additional file 1B, C). GAPDH was used as internal controls in Western blotting analysis. *P < 0.05, **P < 0.01, ***P < 0.001

To investigate the function of ALKBH5 in HCC, we first measured the protein level of ALKBH5 in 67 pairs of patient tumor and adjacent tissues. The results showed that the ALKBH5 protein level was significantly decreased in HCC (Fig. 1A), consistent with the results of previous studies [22]. Herein, we generated ALKBH5 overexpression and knockdown cell lines to investigate the biological functions of ALKBH5 in HCC. CCK8 and EdU incorporation assays were performed to assess the proliferation of the corresponding HCC cell lines. The results showed that overexpression of ALKBH5 inhibited the proliferation of HLF and 97H cells, while knockdown of ALKBH5 in LM3 cells showed the opposite effect (Fig. 1B–E, Additional file 1A). Similarly, the results of the migration, invasion, and wound healing assays indicated that overexpression of ALKBH5 reduced the migration and invasion of HLF and 97H cells, while knockdown of ALKBH5 in LM3 cells showed the opposite effects (Fig. 1F–I, Additional file 1B-C).

### PAQR4 was downregulated by ALKBH5-mediated m6A modification

**Fig. 2:**
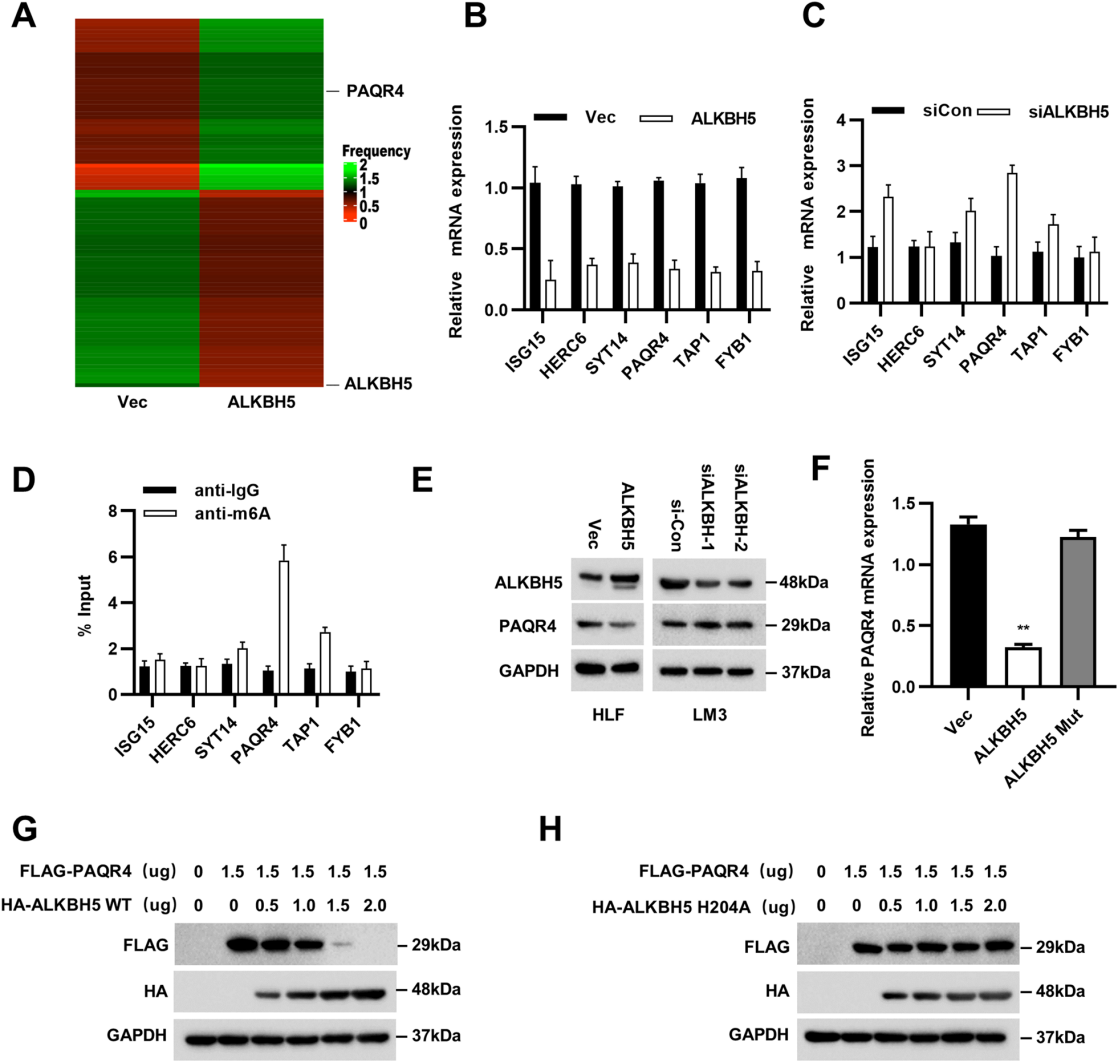
ALKBH5 suppressed m6A modification of PAQR4 mRNA. **A** The heat map exhibited the changed genes of transcriptome sequencing after ALKBH5 overexpression. **B**, **C** RT–qPCR analysis showed the six altered target genes with ALKBH5 knockdown or ALKBH5 overexpression. **D** The result of m6A abundances in mRNA transcripts of ALKBH5 target genes was shown. **E** Western blot analysis showed the protein level of PAQR4 with overexpression of ALKBH5 and knockdown of ALKBH5 in HCC cells. **F–H** RT–qPCR analysis and Western blot verified both mRNA and protein expression of PAQR4 was decreased when co-transfected with ALKBH5-WT but not with ALKBH5-H204A. GAPDH was used as internal controls in Western blotting and RT–qPCR analysis. *P < 0.05, **P < 0.01, ***P < 0.001

To investigate the potential mechanisms by which ALKBH5 inhibits HCC cell proliferation and invasion, mRNA transcriptome sequencing was performed in HLF vector cells and HLF ALKBH5-overexpressing cells. RNA-seq analysis was used to identify possible ALKBH5 target genes (Fig. 2A). We focused on the top 6 downregulated genes: ISG15, HERC6, SYT14, PAQR4, TAP1, and FYB1 (Fig. 2B). Then, we performed RT–qPCR analysis to verify the downregulation of these genes in ALKBH5-overexpressing HLF cells (Fig. 2C). To confirm which genes are regulated by ALKBH5-mediated N6-methyladenosine (m6A) modification, MeRIP assays were performed. The results indicated that the m6A level of PAQR4 was the highest in HLF cells (Fig. 2D). We found that the protein level of PAQR4 was increased after ALKBH5 knockdown but decreased after ALKBH5 overexpression (Fig. 2E). According to a previous study, ALKBH5 H204A exhibited complete loss of demethylase activity relative to that of ALKBH5-WT [16]. After transfection of ALKBH5-WT or ALKBH5-H204A in HEK293T cells, we found that ALKBH5-WT but not ALKBH5-H204A decreased the mRNA level of PAQR4 (Fig. 2F), which indicated that demethylation of ALKBH5 reduced the expression of PAQR4. The results of co-transfection of PAQR4 with either ALKBH5-WT or ALKBH5-H204A in HEK293T cells provided further evidence supporting our conclusion (Fig. 2G–H).

**Fig. 3:**
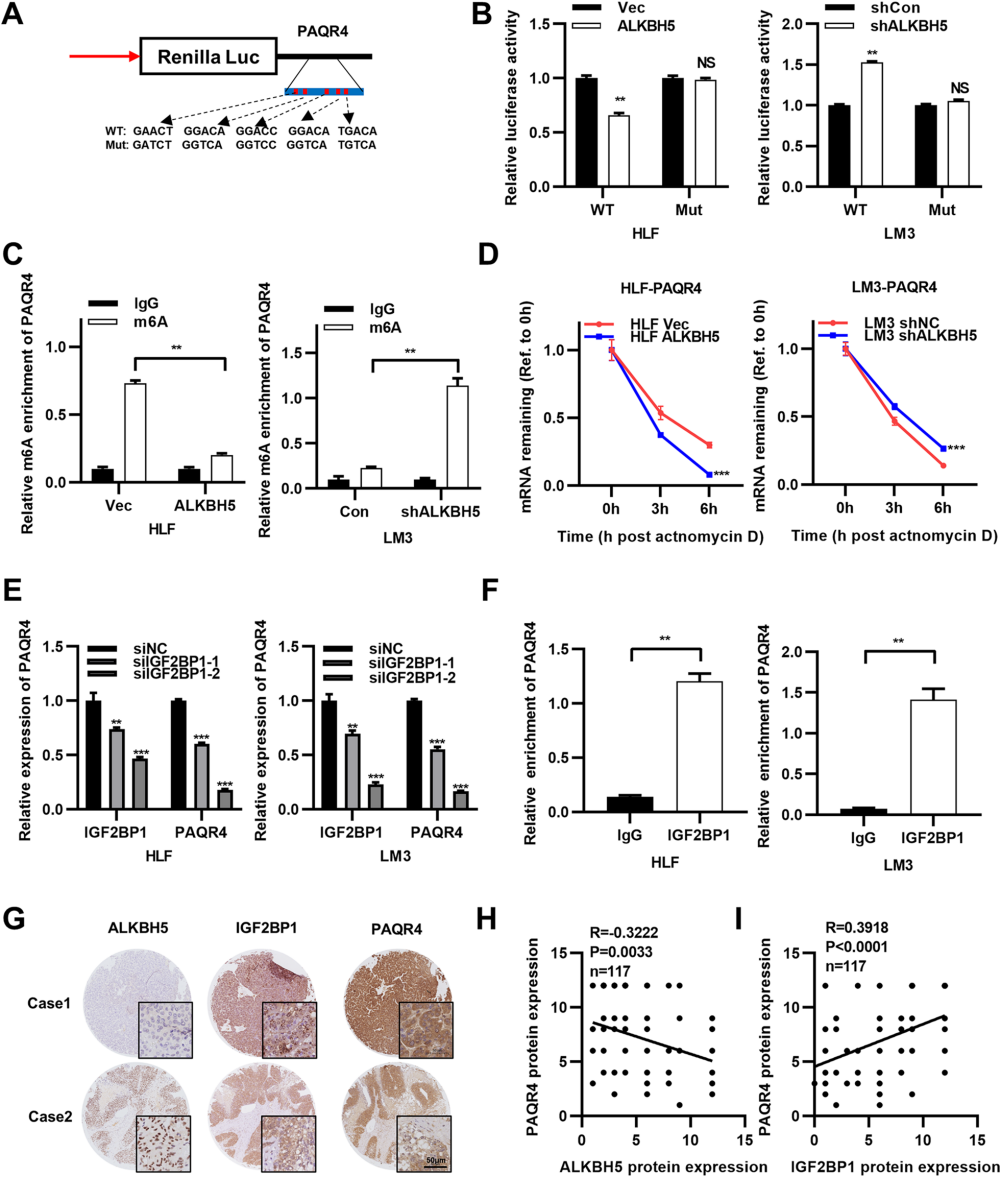
ALKBH5 downregulated PAQR4-mediated with IGF2BP1 in a m6A dependent manner. **A** Schematic representation exhibited totally 5 possible sites of m6A motifs in PAQR4 mRNA, and all of these positions were muted as described then constructed into psiCHECK-Vector plasmids to investigate the m6A roles on PAQR4 expression. PAQR4-WT was constructed into psiCHECK-Vector plasmids as control. **B** The psiCHECK PAQR4-MUT and psiCHECK PAQR4-WT plasmids were transfected into wild-type or ALKBH5-overexpression HLF cells and ALKBH5-knockdown LM3 cells for 24 h. Fluorescence intensity represented changes in PAQR4 transcriptional activity. **C** MeRIP-qPCR analysis indicated ALKBH5 overexpression abolish m6a modification on PAQR4 mRNA in HLF cells while ALKBH5 knockdown enriched m6a modification on PAQR4 mRNA. **D** Actinomycin D (ActD) assay showed overexpression of PAQR4 accelerate the degradation of mRNA whereas this process slowed down after ALKBH5 was knockdown. **E** PAQR4 expression was measured via RT–qPCR in two IGF2BP1 knockdown HCC cells. **F** The result of RIP-qPCR indicated that IGF2BP1 could bind to PAQR4 mRNA in HLF and LM3 cells. GAPDH was used as internal controls in qPCR analysis. **G** Representative immunohistochemical staining images of ALHBH5, IGF2BP1 and PAQR4 in human HCC tissues; scale bar: 200 μm. **H** Correlation analysis between ALHBH5 and PAQR4 in human HCC tissues. **I** Correlation analysis between IGF2BP1 and PAQR4 in human HCC tissues. *P < 0.05, **P < 0.01, ***P < 0.001

We constructed the psiCHECK-PAQR4 luciferase reporter plasmid to detect the effect of m6A modification on PAQR4 expression. To construct the mutant forms of PAQR4, all five predicted methylation regions in the PAQR4 CDS and its 3′UTR were replaced as shown in the Fig. 3A. Then, the PAQR4-WT or PAQR4-MUT plasmid was co-transfected with ALKBH5 or si-ALKBH5. The results indicated that the translation efficiency of PAQR4 was negatively correlated with the expression of ALKBH5 (Fig. 3B). MeRIP-qPCR analysis was then performed to reveal the correlation between the protein level of ALKBH5 and the m6A level of PAQR4. The m6A level of PAQR4 decreased when ALKBH5 was overexpressed. Conversely, when ALKBH5 was knocked down, the m6A level of PAQR4 increased (Fig. 3C). Furthermore, we discovered that overexpression of ALKBH5 reduced the PAQR4 mRNA degradation rate, whereas knockdown of ALKBH5 accelerated this process (Fig. 3D). In our research, it was shown that ALKBH5 expression was related to the decreased m6A methylation level of PAQR4, which led to decreased PAQR4 mRNA stability. Previous research has shown that m6A “readers” in the YTHDF and IGF2BP families might play key roles in mRNA methylation and in mRNA stability, translation, and/or localization [24]. Here, we discovered that when IGF2BP1 was knocked down, the mRNA level of PAQR4 was reduced (Fig. 3E). This finding was consistent with the role of IGF2BP1 proposed in previous reports [25]. We then used a RIP assay and observed that PAQR4 mRNA was enriched in IGF2BP1-overexpressing cells, implying that PAQR4 mRNA may be degraded through interaction with IGF2BP1 (Fig. 3F). To validated that ALKBH5 and IGF2BP1 are really relevant with PAQR4, correlation analyses were performed to show that ALKBH5 was negative correlated with PAQR4 and IGF2BP1 was positive correlated with that of PAQR4 in the HCC samples (Fig. 3G–I). In summary, PAQR4 was downregulated by ALKBH5-mediated m6A modification in an IGF2BP1-dependent manner.

### PAQR4 promoted HCC cell proliferation in vivo and in vitro

**Fig. 4:**
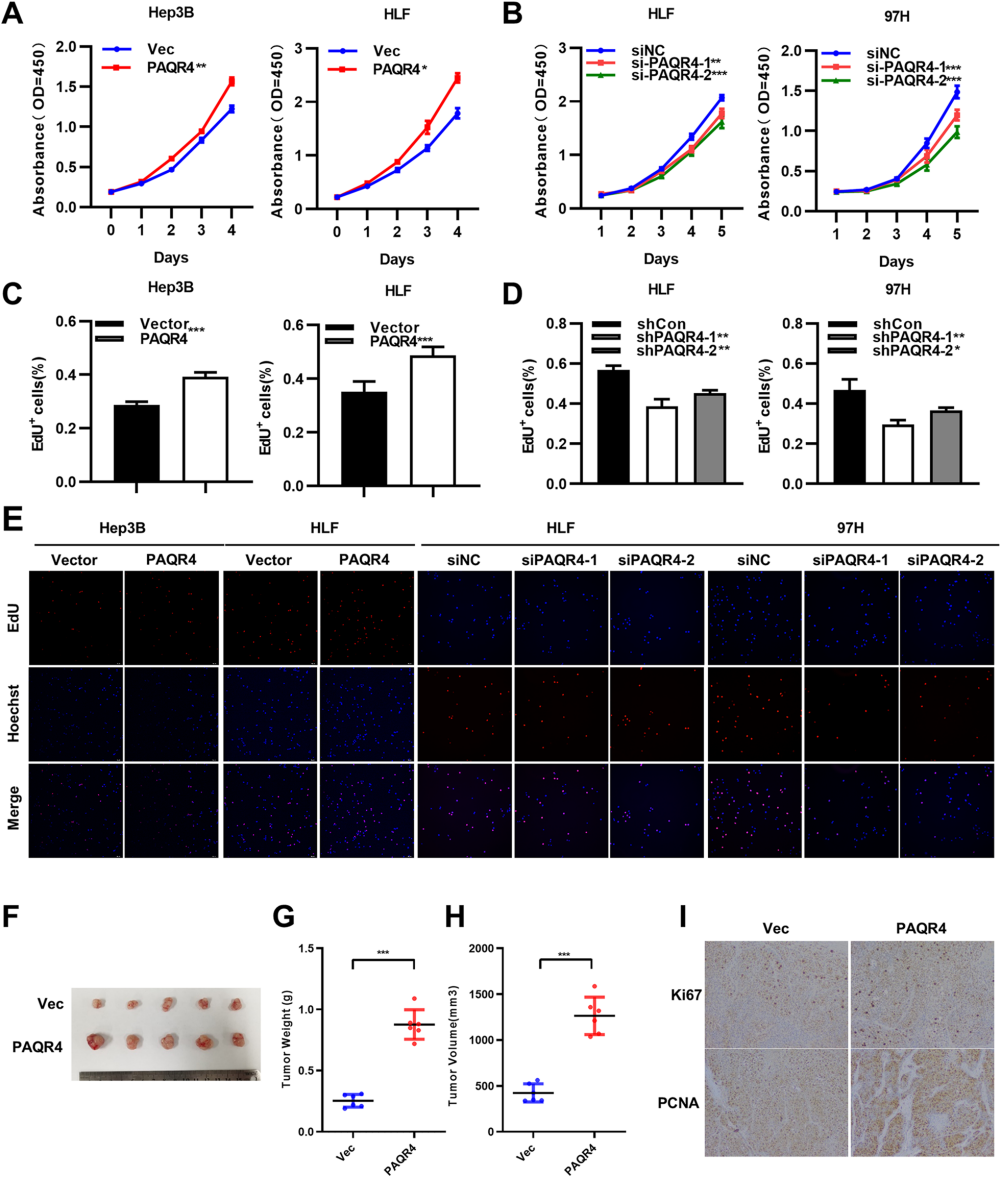
PAQR4 promoted HCC cells proliferation in vivo and in vitro. CCK8 and EdU assays confirmed that overexpression of PAQR4 promote proliferation in Hep3B and HLF cells (**A**, **C** and **E**), whereas knockdown of ALKBH5 repressed cell proliferation in HLF and 97H cells (**B**, **D** and **E**). **F** Representative images of tumor xenograft models using PAQR4-overexpressing HLF cells (n = 5), HLF-Vector cells were used as control. (**G** and **H**) Tumor weight and volume of PAQR4-overexpressing and control group were shown. **I** The Immunohistochemistry (IHC) assays showed expression of ki-67 and PCNA in the tumor xenograft model. Scale bar, 20 μm (40 ×). *P < 0.05, **P < 0.01, ***P < 0.001

To investigate the biological function of PAQR4 in HCC, PAQR4 was overexpressed in Hep3B and HLF cells and knocked down in HLF and 97H cells. The CCK8 and EdU incorporation assays showed that overexpression of PAQR4 promoted the proliferation of Hep3B and HLF cells and that knockdown of PAQR4 suppressed the proliferation of HLF and 97H cells (Fig. 4A–E). In vivo experiments were also completed to demonstrate these findings (Fig. 4F). The tumor weight and tumor volume in the PAQR4 overexpression group were substantially higher than those in the control group in the subcutaneous xenograft model (Fig. 4G and H). Correspondingly, PAQR4 overexpression was associated with higher expression levels of the proliferation indices Ki-67 and PCNA, according to immunohistochemistry (IHC) (Fig. 4I).

### PAQR4 promoted the migration and invasion of HCC cells in vitro and promoted HCC metastasis in vivo

**Fig. 5:**
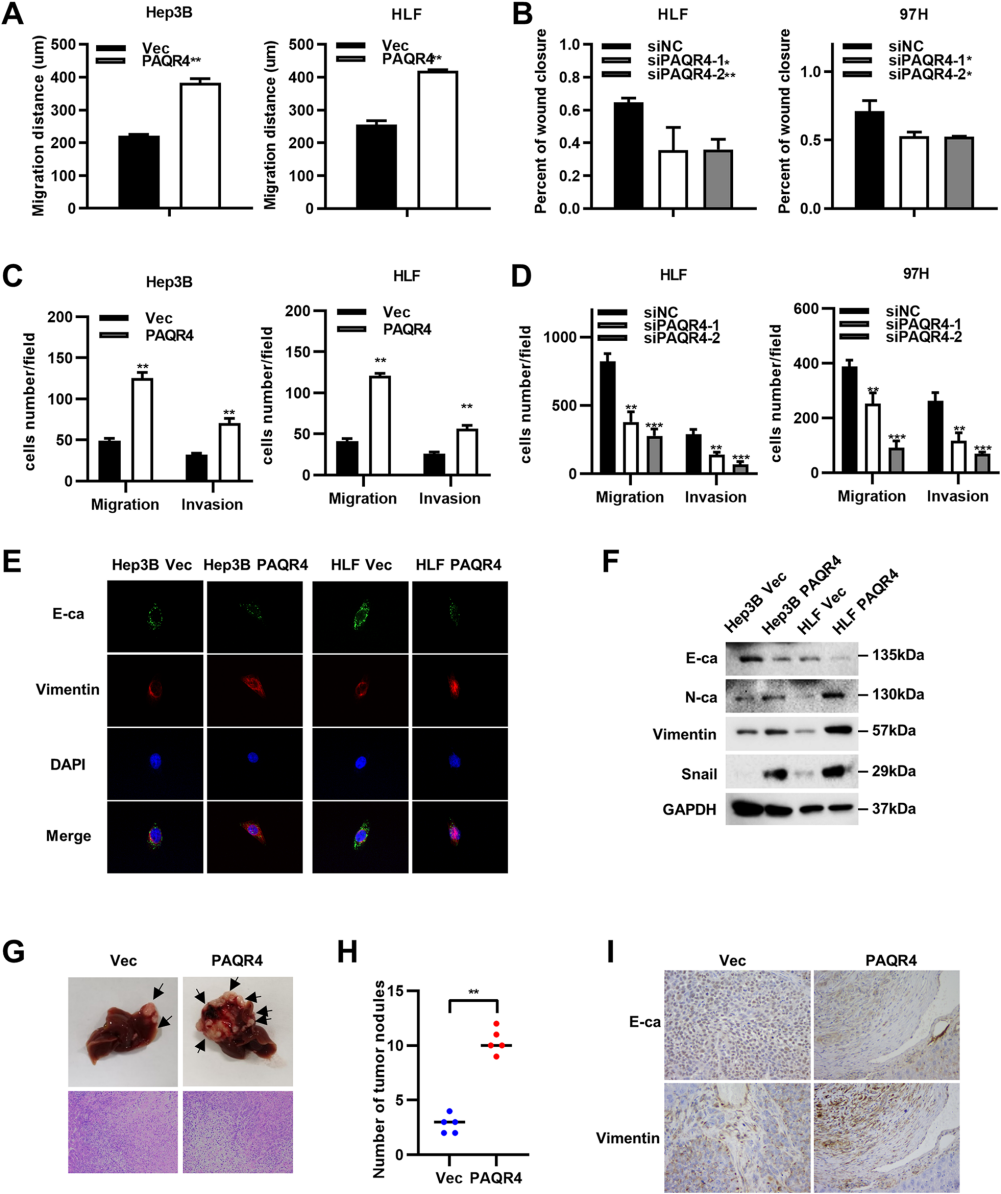
PAQR4 promoted the migration and invasion capability of HCC cells in vitro and promoted HCC metastasis in vivo. Transwell and wound healing assays showed that overexpression of PAQR4 promote the capability of migration and invasion in Hep3B and HLF cells (**A**, **C** and Additional file 2A and B). Knockdown of PAQR4 suppress the capability of migration and invasion in HLF and 97H cells (**B, D** and Additional file 2A and B). Immunofluorescence and Western blot analysis EMT markers like E-ca, N-ca, Vimentin in Hep3B and HLF cells (**E**, **F**). Representative images of intrahepatic metastasis model using PAQR4-overexpressing HLF cells (n = 5), HLF-Vector cells were used as control (**G**). The numbers of metastasis nodules (**H**). The Immunohistochemistry (IHC) assays showed expression of EMT markers E-ca and N-ca on tumor sections. Scale bar, 20 μm (40 ×). **i** *P < 0.05, **P < 0.01, ***P < 0.001

The wound healing, migration, and invasion assays showed that PAQR4 promoted the migration and invasion of Hep3B and HLF cells and that knockdown of PAQR4 suppressed the migration and invasion of HLF and 97H cells (Fig. 5A–D and Additional file 2A, B). Since epithelial-mesenchymal transition (EMT) is of great importance in the migration and invasion of HCC cells [26], we hypothesized that PAQR4 may promote migration and invasion by stimulating EMT signaling. Our data obtained from immunofluorescence staining and Western blot analysis indicated that the epithelial marker E-cadherin was upregulated in PAQR4-overexpressing Hep3B and HLF cells, while the mesenchymal marker vimentin was downregulated in PAQR4-overexpressing Hep3B and HLF cells (Fig. 5E and F). Consistent with these findings, the staining results in tumor tissues also showed the same trend (Fig. 5I). Collectively, these findings suggested that overexpression of PAQR4 activates EMT in HCC. Moreover, overexpression of PAQR4 significantly increased the formation of intrahepatic metastatic foci compared to that in the control group in the intrahepatic HCC implantation model (Fig. 5G and H). Taken together, these results suggested that overexpression of PAQR4 promotes the metastasis of HCC cells.

To further confirm that the protective effect of ALKBH5 could be blocked by the overexpression of PAQR4, corresponding functional rescue assays were performed. As EdU and CCK8 assays showed, overexpression of ALKBH5 decreased the proliferation capacity in 2 HCC cell lines, while co-overexpressed of PAQR4 reverted this phenomenon (Additional file 3A and B). Wound healing and transwell assays showed that overexpression of ALKBH5 decreased the invasion and migration ability, while co-overexpressed of PAQR4 reverted this phenomenon (Additional file 3C and D). To sum up, the protective effect caused by upregulation of ALKBH5 in HCC cell lines may achieved by reducing the effect of PAQR4.

### PAQR4 promoted the development of HCC by activating AKT

**Fig. 6:**
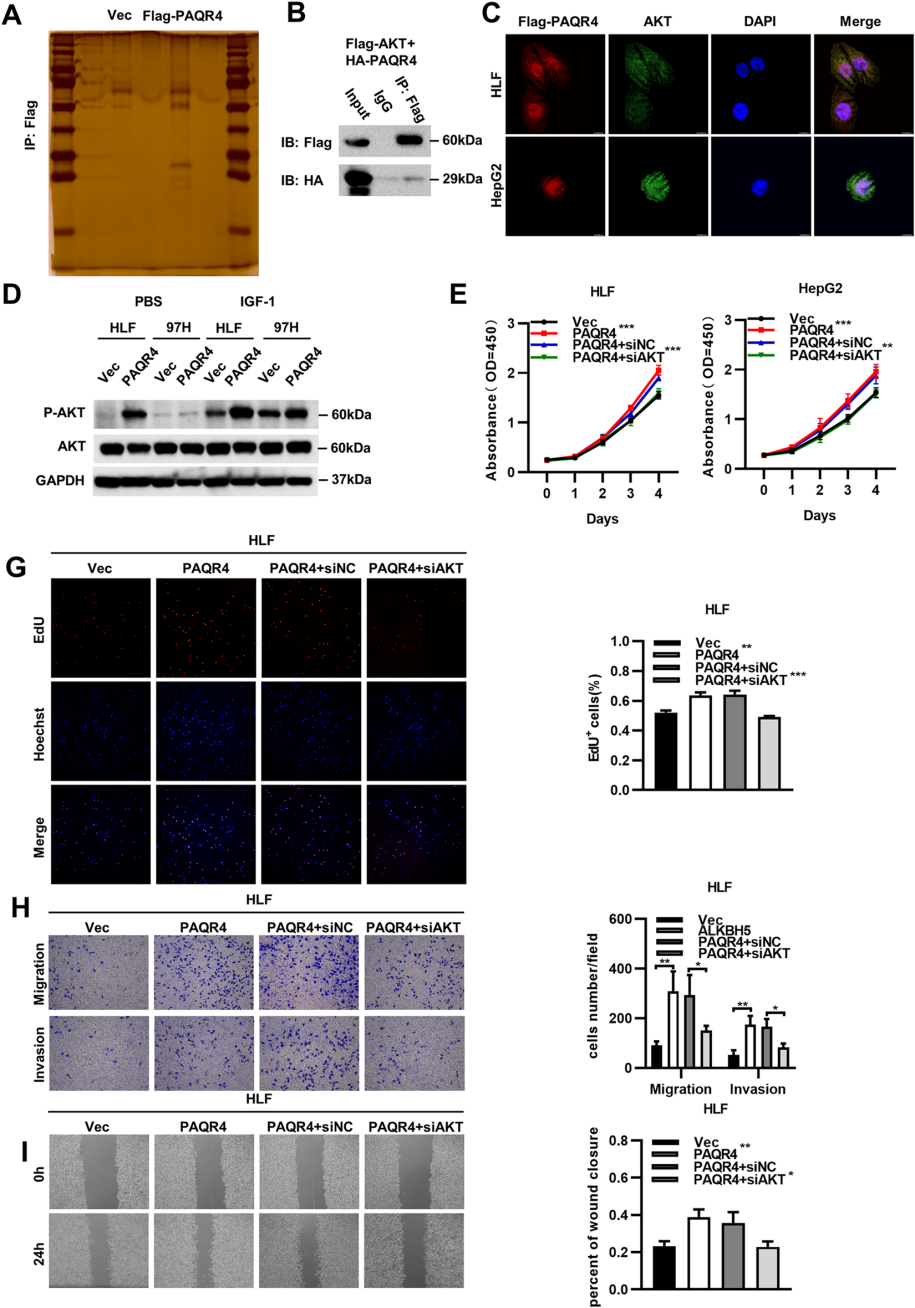
PAQR4 promoted the development of HCC by activating AKT. **A** Silver staining indicated PAQR4 may interact with AKT. **B** Co-immunoprecipitation (Co-IP) assay identified the interaction between PAQR4 and AKT. **C** The colocalization of PAQR4 and AKT was observed by immunofluorescence in HLF and HepG2 cells. **D** Western blot analysis showed activation of AKT pathway with or without IGF-1 stimulation when PAQR4 was overexpressed. (**E**, **F** and Additional file 4A) CCK8 and EdU assays showed knockdown of AKT reduce proliferation caused by overexpression of PAQR4. (**G**, **H** and Additional file 4B, C) Transwell and wound healing assays showed that knockdown of AKT reduce increased migration and invasion capability caused by overexpression of PAQR4. GAPDH was used as internal controls in Western blotting analysis. *P < 0.05, **P < 0.01, ***P < 0.001

To further explore how PAQR4 promotes the development of HCC, silver staining and subsequent mass spectrometry were performed to identify potential binding partners of PAQR4 (Fig. 6A). Interestingly, we found that the characteristic peptides of AKT were identified by mass spectrometry. In line with this result, we conducted exogenous co-IP assays in HEK293T cells (Fig. 6B) and performed an immunofluorescence assay, thus showing the colocalization of PAQR4 and AKT in the cytoplasm (Fig. 6C). The results of both of these assays suggested an interaction between PAQR4 and AKT. In previous studies, it has been shown that PAQR4 promotes tumorigenesis and metastasis via the PI3K/AKT pathway in hepatocellular carcinoma and prostate cancer [27, 28]. We detected the activation of AKT in wild-type and PAQR4-overexpressing HCC cells stimulated with or without Insulin-like growth factor-1 (IGF-1), which is regarded as an activator of the AKT pathway [29, 30]. The Western blot analysis results revealed that PAQR4 overexpression increased the phosphorylation of AKT regardless of IGF-1 stimulation (Fig. 6D). We also knocked down AKT in PAQR4-overexpressing HCC cells to evaluate cell proliferation, migration, and invasion. Knockdown AKT significantly prevented the increases in proliferation, migration, and invasion induced by PAQR4 overexpression in HCC cells (Fig. 6E–I, Additional file 4A–C). Taken together, these data suggested that PAQR4 enhanced proliferation, migration, and invasion by activating AKT in HCC cells (see Additional file 5).

### High expression of PAQR4 is associated with poor prognosis in HCC

**Fig. 7:**
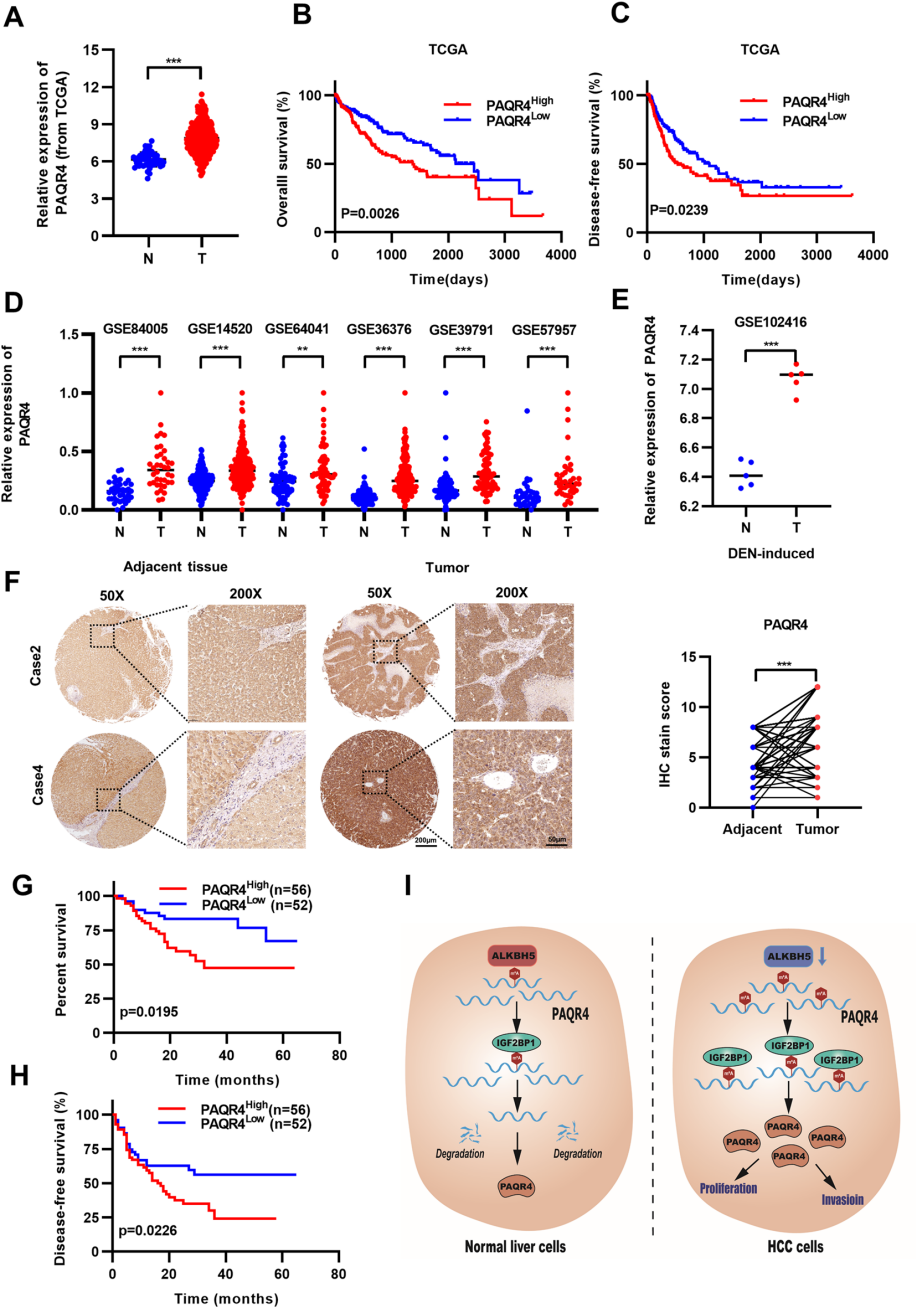
High expression of PAQR4 is associated with poor prognosis in HCC. **A** PAQR4 expression in the normal and HCC data were obtained from TCGA and the (**B**, **C**) Kaplan–Meier survival curves disease-free survival of patients with HCC from TCGA. **D** PAQR4 expression in human HCC data were obtained from 6 GEO datasets. **E** PAQR4 expression in DEN-induced mouse liver cancer. **F** Representative images of Immunohistochemistry (IHC) from human HCC samples and the expression of PAQR4 was compared between Adjacent tissue and tumor tissue according to the IHC scoring. **G**, **H** Kaplan–Meier survival curves and disease-free survival of patients with HCC from Tongji cohort. **i** Schematic representation of a model for the role of PAQR4 in HCC. *P < 0.05, **P < 0.01, ***P < 0.001

To explore the potential implications of PAQR4 in the prognosis of patients with HCC, we elected to perform studies of PAQR4 data from multiple databases. The data from The Cancer Genome Atlas (TCGA) database indicated that the PAQR4 level was significantly increased in tumor tissues (Fig. 7A). In the same database, the results of Kaplan–Meier analysis revealed that high expression of PAQR4 was significantly correlated with shorter OS and DFS times (Fig. 7B, C). In addition, in six different GSE datasets, PAQR4 was found to be highly expressed in HCC patients (Fig. 7D). Interestingly, our study showed that a higher expression level of PAQR4 was detected in HCC tumor tissues than in the adjacent normal tissues in the diethylnitrosamine (DEN)-induced mouse model (Fig. 7E). IHC staining of a total of 108 pairs of clinical HCC tissues demonstrated that the expression of PAQR4 was significantly increased in tumor tissues (Fig. 7F). Patients with higher PAQR4 expression levels exhibited worse prognoses, and the same was true in our cohort (Fig. 7G, H). These clinical data supported our finding that high expression of PAQR4 was associated with poor prognosis in HCC. Together, our results demonstrated that ALKBH5- mediated demethylation of PAQR4 mRNA resulted in downregulation of PAQR4. This study also implies that PAQR4 plays an important role in HCC development and is an important biomarker for poor prognosis in HCC (Fig. 7I).

## Discussion

For decades, an increasing number of studies have shown that m6A mRNA modification affects the biology of normal and tumor cells [31–34]. STM2457, a new bioavailable inhibitor of METTL3, was developed in 2021 as a new drug to treat myeloid leukemia [35]. As the first methylase-targeted anticancer drug, it was proven to be effective and safe in vivo, constituting an example of successfully using m6A as a therapeutic target [36]. In the present study, we found that the expression of ALKBH5 was decreased in HCC. Overexpression of ALKBH5 suppressed the proliferation, migration, and invasion of HCC cells in vitro*,* while knockdown of ALKBH5 exhibited the opposite effects. As shown in our previous publication [22], in vivo experiments to demonstrate the antitumor effect of ALKBH5 were not repeated in this study. The data from transcriptome analyses inspired us to search for possible downstream targets of ALKBH5. After verification of mRNA and protein levels, we established PAQR4 as our research target of an ALKBH5 substrate molecule. Subsequently, we observed that the effect of ALKBH5 on PAQR4 was dependent on the activity of the enzyme and was abolished by the ALKBH5 H204A mutation, which suggests that the function of ALKBH5 was mediated through m6A modification. The results of MeRIP analysis and the luciferase reporter assay also confirmed this conclusion. After identifying the eraser of m6A in PAQR4 mRNA and clarifying its effects on PAQR4, we next looked for the m6A reader, which can “recognize” PAQR4 mRNA methylation. YTH domain-containing proteins and insulin-like growth factor proteins function as m6A readers to regulate mRNA stability and translation [12, 24, 37–40]. Surprisingly, we found that IGF2BP1 bound to PAQR4 mRNA and prevented its degradation. However, other potential m6A readers of PAQR4 have not been identified and require further investigation.

Progestin and AdipoQ Receptor 4 (PAQR4) belongs to the progestin and adipoQ receptor family. This family includes 11 proteins (PAQR1 to PAQR11) that encode functional receptors, as their names suggest [41]. In recent studies, PAQR4 was demonstrated to act as an oncogene in multiple cancers. PAQR4 was discovered to disrupt the interaction between Nrf2 and Keap1 in non-small-cell lung cancer (NSCLC), preventing Nrf2 protein degradation and thereby decreasing the sensitivity of malignant cells to chemotherapy [42]. PAQR4 inhibits SKP2-mediated ubiquitination of CDK4 by competitive binding to the binding site, contributing to cancer cell proliferation and carcinogenesis [43]. In breast cancer, PAQR4 was discovered to be located in the Golgi apparatus, where it reduces cytotoxicity and generates S1P to aid cell development [44]. In HCC, PAQR4 promotes the development of HCC through the PI3K/AKT pathway [27], as also shown in our study. In this research, we demonstrated that PAQR4 promotes the proliferation, invasion, and metastasis of HCC cells in vivo and in vitro. We found that the expression of PAQR4 was significantly upregulated in our patient cohort and in TCGA data. In the diethylnitrosamine (Den)-induced mouse model of liver cancer, we also observed high expression levels of PAQR4 and found that it was correlated with shorter overall survival and disease-free survival rates. These data indicate that PAQR4 might be used as a prognostic factor in HCC patients.

Overall, AKT signaling has many biological functions in normal and tumor cells [29, 45]. Particularly in HCC, AKT is regarded as a “driver” of cancer development and malignant behaviors [46, 47]. In this study, we found that overexpression of PAQR4 facilitated but knockdown of PAQR4 counteracted the activation of AKT. In addition, we knocked down AKT in PAQR4-overexpressing cancer cells in vitro and found that the proliferation, invasion, and migration of these cells were restored. Based on our existing data, we concluded that PAQR4 may exert its tumor-promoting effects by activating the AKT pathway in HCC. This conclusion may be further supported by using the PI3K/AKT signaling-specific inhibitor LY294002 in mice in future experiments.

## Conclusions

In our studies, we found that ALKBH5 inhibits HCC growth by downregulating PAQR4 expression in an IGF2BP1-dependent manner. Furthermore, we found that PAQR4 enhanced proliferation, migration, and invasion by activating AKT in HCC cells.

## Methods

### Clinical samples or patients and specimens

All HCC specimens and paired adjacent normal tissues were collected from patients who underwent curative surgical resection between 2012 and 2017 at the Hepatic Surgery Center, Tongji Hospital of Huazhong University of Science and Technology (HUST; Wuhan, China). A set of 108 pairs of HCC and adjacent normal tissues were used for immunohistochemistry (IHC) and prognostic analysis. Another 80 pairs of HCC and adjacent normal tissues were used for Western blotting (WB) to detect the expression of PAQR4. Informed consent for the use of human material was obtained from each patient, and the study was approved by the Ethics Committee of Tongji Hospital of HUST.

### Animal models

The animal experimental protocols and ethical considerations were approved by the Institutional Animal Care and Treatment Committee of Huazhong University of Science and Technology. Four-week-old male BALB/c nude mice were purchased from HFK BioTechnology and maintained under specific pathogen-free conditions. For the subcutaneous xenograft model, 1 × 10^6^ tumor cells were resuspended in 100 μL of DMEM and then injected into the axilla of each mouse (n = 5 mice per group). The mice were sacrificed after 2 weeks, the tumor weight was recorded, and the tumor volume was calculated according to the following equation: volume = 1/2*(length × width2). After the experiment, the specimens were fixed with 4% formaldehyde. For the intrahepatic tumor implantation model, 1 × 10^6^ tumor cells were resuspended in 100 μL of DMEM and then inoculated under the capsule of the right lobe of the liver (n = 5 mice per group). The mice were sacrificed after 4 weeks, and the tumors were removed and subjected to HE staining.

### Cell lines and cell culture

The HCC cell lines LM3, HepG2, and HEK293T were purchased from the China Center for Type Culture Collection (Wuhan, China), and the HCC cell lines HLF, Hep3B, and 97H were obtained from the Hepatic Surgery Center, Tongji Hospital of Huazhong University of Science and Technology (HUST; Wuhan, China). All of the above cells were cultured in Dulbecco’s modified Eagle’s medium (DMEM, Gibco, Grand Island, NY) supplemented with 10% fetal bovine serum (FBS; Gibco, Grand Island, NY) and incubated in 5% CO_2_ at 37 °C.

### In vitro cell proliferation assays

An EdU Cell Proliferation Kit (Beyotime) was used to evaluate cell proliferation in vitro. Cells were plated into 24-well plates at a density of 4.0 × 10^5^ cells per plate. After adherent growth, EdU (10 μM) was added to the cells and incubated at 37 °C for 1 h. Then, PBS was used to remove the DMEM and preserve the EdU probe. Cells were fixed with 4% formaldehyde for 30 min. After being washed twice with PBS, the cells were permeabilized in 0.3% Triton X-100 for 10 min. Click Additive Solution was then added to the cultured cells and incubated at room temperature in the dark for 30 min. Nuclei were stained with Hoechst for 10 min. Images were acquired by microscopy.

### Colony formation assay

HCC cells were seeded into 6-well plates (200 cells/well) and further cultured at 37 °C for 2 weeks. The cells were then fixed with 4% formaldehyde for 30 min and stained with 0.1% crystal violet for 15 min. Then, the colonies were counted.

### CCK8 assay

The in vitro HCC cell viability assay was conducted using a Cell Counting Kit-8 (CCK8). Cells were seeded into 96-well plates (100 cells/well) at 37 °C in 5% CO_2_. Then, 100 µl of CCK8 solution diluted tenfold with DMEM was added to each plate and incubated at 37 °C for 1 h. Cell proliferation activity was then evaluated with a microplate reader.

### Migration and invasion assays

According to the manufacturer’s protocol, Transwell migration and invasion assays were performed using a 24-well Transwell plate containing membranes with 8-μm pores (Costar, Cambridge, MA). For the migration assay, 3 × 10^4^ cells resuspended in 100 µl of serum-free DMEM were seeded in the upper chambers, while 700 µl of DMEM containing 10% fetal bovine serum (FBS) was placed in the lower chambers. For the invasion assay, the membranes in the upper chambers were first coated with 50 μL of a 1:4 mixture of Matrigel (BD Biosciences, USA): DMEM. Then, 3 × 10^4^ cells resuspended in 100 µl of serum-free DMEM were seeded in the upper chambers, while 700 µl of DMEM containing 10% FBS was placed in the lower chambers. After culture for 24 h in 5% CO_2_ at 37 °C, the cells that passed through the lower surface of the membranes were fixed with 4% formaldehyde for 30 min at room temperature and then stained with 0.1% crystal violet at room temperature for 15 min. The cells were imaged by bright-field microscopy and then counted with ImageJ software (NIH open source image processing software). Each experiment was repeated at least 3 times.

### Wound healing assay

A wound healing assay was used to evaluate the cell migration ability in vitro. HCC cells were seeded in 6-well plates at 90% to 100% confluence. Sterilized 10 μL pipette tips were used to scratch across the surface of the plate to create an artificial wound. The cells were then washed with PBS 3 times to remove detached cells and cell debris. This step was followed by imaging using a bright-field microscope 0 h and 24 h after the scratch was made. Each experiment was repeated at least 3 times.

### Western blotting (WB)

Cells were lysed with RIPA buffer containing protease inhibitor cocktail and phosphatase inhibitor cocktail (MedChemExpress, USA) at 4 °C for 30 min. Protein concentrations in the cell lysates were quantified by using a BCA Protein Assay Kit (Thermo Fisher Scientific). Briefly, equal amounts of protein were then separated by SDS–PAGE (Boster Biological Technology, Wuhan, China) and transferred to polyvinylidene fluoride (PVDF) membranes (Millipore, USA). The membranes were blocked with 5% bovine serum albumin (BSA) at 37 °C for 60 min and then incubated with primary antibodies at 4 °C overnight. Next, the membranes were incubated with goat anti-rabbit or goat anti-mouse immunoglobulin as secondary antibodies at 37 °C for 1 h. Finally, the membranes were visualized with an ECL detection system (Bio-Rad Laboratories). Image Lab™ 4.0 software was used to analyze the Western blot band densities, and GAPDH was used as the control for normalization to the protein content.

### Antibodies and reagents

Primary antibodies against PAQR4 (13401-1-AP for WB and IHC), GAPDH (60004-1-Ig for WB), and ALKBH5 (16837–1-AP for WB) were purchased from Proteintech (Wuhan, Hubei, China). Primary antibodies against Flag (F1804 for WB and IP) and HA (H6908 for WB and IP) were purchased from Sigma (St. Louis, MO, USA). Primary antibodies against AKT (#4691 for WB and IF), phospho-Akt (Ser473) (#4060 for WB), Vimentin (#5741 for WB, IF and IHC), and Snail (#3879 for WB) were purchased from CST (Danvers, MA, USA). Primary antibodies against E-cadherin (#610182 for WB, IF and IHC) and N-cadherin (#610920 for WB) were purchased from BD Biosciences (NJ, USA). Primary antibody against IGF2BP1 (#A1517 for IHC) was purchased from ABclonal (Wuhan, Hubei, China). Primary antibody against ALKBH5 (#ab195377 for IHC) was purchased from Abcam Technology.

### Coimmunoprecipitation (co-IP)

Cells were lysed in IP lysis buffer containing protease inhibitor cocktail and phosphatase inhibitor cocktail (MedChemExpress, USA) at 4 °C for 30 min. Afterward, the cell lysates were immunoprecipitated with the indicated antibodies at 4 °C overnight. On the second day, the lysates were incubated with Magnetic Agarose Beads (Biolinkedin, Shanghai, China) for 1 h prior to 3 washes with Binding/Washing buffer (0.5 M NaCl and 20 mM Na_2_HPO_4_, pH 7.0). The washed beads were eluted with 2 × loading buffer and then subjected to Western blot analysis. The protein interacting with PAQR4 was screened by immunoprecipitation, silver staining and mass spectrometry. Cell extracts from 293 T cells transfected with Flag-tagged PAQR4 or Flag-tagged vector were immunoprecipitated with anti-Flag antibody. The corresponding IP experiment steps were described above and samples were carried out silver staining to ensure samples quality. Then eluted proteins were identified by mass spectrometry.

### Immunofluorescence (IF) analysis

A total of 3 × 10^5^ cells were seeded into glass dishes, and 8 h later, the cells were fixed with 4% formaldehyde for 15 min at room temperature. Then, the cells were washed 3 times and permeabilized with 0.1% Triton X-100 for 30 min. After blocking with 5% bovine serum albumin (BSA) at 37 °C for 60 min, the cells were incubated with a primary antibody overnight at 4 °C. The next day, the cells were washed three times and incubated with a fluorescent secondary antibody for 2 h at room temperature in the dark. Finally, nuclei were stained with 4′,6-diamidino-2-phenylindole (DAPI; Sigma–Aldrich) for 8 min. Images were acquired with a laser scanning confocal microscope.

### Immunohistochemistry (IHC)

Tumor tissues were fixed with 4% formaldehyde for 1 day at room temperature, embedded in paraffin and sliced into sections. The paraffin sections were first warmed to 65 °C for 30 min and then placed sequentially into xylene, xylene, 100% alcohol, 100% alcohol, 95% alcohol, 90% alcohol, 80% alcohol, and 70% alcohol for dewaxing. Then, the slices were heated to 95 °C for 30 min for antigen repair. After the slices were cooled to room temperature and then washed 3 times with PBS, they were immersed in 3% H_2_O_2_ for 10 min to quench endogenous peroxidase activity. The slices were blocked with 5% bovine serum albumin at 37 °C for 60 min and then incubated with primary antibodies at 4 °C overnight. On the second day, after being washed 3 times with PBS, the slices were incubated with a horseradish peroxidase (HRP)-conjugated secondary antibody for 1 h. Finally, DAB (Dako; Agilent Technologies, Inc.) was used to visualize staining. Staining was observed under a microscope after hematoxylin counterstaining, dehydration, and sealing of the slices. Immunohistochemical staining scoring of human HCC samples was performed by two pathologists in double blind. In each example, five visual fields were examined under a microscope, and scores were assigned based on the number of positive cells and the staining intensity. If fewer than 5% of the total cells are stained, the number of positive cells receives a score of 0, 26% to 50%, 51% to 75%, and 76% to 100%, respectively. According to the staining intensity standard, 0 is colorless, 1 is pale yellow, 2 is brown, and 3 is brown. The final staining score is the product of two scores.

### RNA extraction and RT–qPCR

RNA was extracted by using a FastPure Cell/Tissue Total RNA Isolation Kit (Vazyme) following the manufacturer’s instructions. cDNA libraries were prepared by using HiScript II Q Select RT SuperMix for qPCR (Vazyme). Then, the number of transcripts was quantified with the CFX96 Touch™ Real-Time PCR Detection System (Bio-Rad, Hercules, CA, USA) by using ChamQ SYBR qPCR Master Mix (Vazyme) following the manufacturer’s instructions. The amounts of target gene transcripts were normalized to the internal gene GAPDH. Each experiment was repeated at least 3 times. The All primers used were as follows: GAPDH-F, 5′-GACAAGCTTCCCGTTCTCAG-3′; GAPDH-R, 5′-GAGTCAACGGATTTGGTCGT-3′; PAQR4-F, 5′-FTACCTGCACAACGAACTGGG-3′; PAQR4-R, 5′ -AAGAGGTGATAGAGCACGGAG-3′; ISG-15-F, 5′ -CGCAGATCACCCAGAAGATCG-3′; ISG-15-R, 5′ -TTCGTCGCATTTGTCCACCA-3′; HERC6-F, 5′ -CCCTCAGTGGGCGTAATGTC-3′; HERC6-R, 5′ -AGAGCGATTGTCTCCAAATGTG-3′; SYT14-F, 5′ -AAATACAGTCCTCTATCGGCAGA-3′; SYT14-R, 5′ -TTGGGCACTTGTTATATGAGCAT-3′; TAP1-F, 5′ -CTGGGGAAGTCACCCTACC-3′; TAP1-R, 5′ -CAGAGGCTCCCGAGTTTGTG-3′; FYB1-F, 5′ -GCAGGCCAAAGATTCGGAAC-3′; FYB1-R, 5′ -GGAGGCCAGGGAAATGTAGG-3′; ALKBH5-F, 5′ -CGGCGAAGGCTACACTTACG-3′; ALKBH5-R, 5′ -CCACCAGCTTTTGGATCACCA-3′; IGF2BP1-F, 5′ -GCGGCCAGTTCTTGGTCAA-3′; IGF2BP1-R, 5′ -TTGGGCACCGAATGTTCAATC-3′.

### Plasmid construction and transfection

The human PAQR4 or ALKBH5 sequence was tagged with Flag or HA at the N-terminus and inserted into the pcDNA3.1 vector (Invitrogen, USA). Short hairpin RNAs (shRNAs) targeting PAQR4 and ALKBH5 were inserted into the pcDNA3.1 vector to construct knockdown plasmids. The ALKBH5 H204A sequence was constructed by site-directed mutagenesis. Human PAQR4 and ALKBH5 were also inserted into the pLenti vector (Invitrogen, USA) to construct overexpression plasmids. All the above plasmids were confirmed by DNA sequencing. A lentiviral system was used to construct cell lines with stable knockdown and overexpression. Small interfering RNA (siRNA) oligos were designed by RiboBio (Guangzhou, China) for gene silencing. Lipo3000 (Invitrogen) was used in all of the above transfections according to the manufacturer’s recommendations. The sequences of the siRNAs are summarized as follows: PAQR4 si-1, GCAGGCTCCGTGCTCTATCAC; PAQR4 si-2, CGTCTTGCTCTGAGAGTTCAA; ALKBH5 si-1, GCTGCAAGTTCCAGTTCAA; ALKBH5 si-2, CCTCAGGAAGACAAGATTAGA; IGF2BP1 si-1, GGCTCAGTATGGTACAGTA; IGF2BP1 si-2, TGAAGATCCTGGCCCATAA.

### RNA immunoprecipitation (RIP) assays

Cells were washed twice with precooled PBS and then digested with RIP buffer. After ultrasonic lysis and centrifugation at 4 °C for 15 min, 20 µL of each sample was removed as input. Different antibodies were added separately to the remaining samples, and IgG was also added as the negative control. After overnight incubation at 4 °C, magnetic beads were added to each sample. After magnetic beads were added, the samples were incubated at 4 °C for 4 h. After 4 washes with buffer, TRIzol was added to the tube to extract RNA. RNA was reverse transcribed into cDNA for subsequent experiments.

### Statistical analysis

Statistical analyses of all data were carried out using GraphPad Prism 8.3.0 (GraphPad, La Jolla, CA, USA) software. All values are expressed as the means ± SEMs. Comparisons between two groups were performed by two-tailed Student’s t test or one-way or two-way ANOVA. The Kaplan–Meier method with the log-rank test was used to assess survival in different subgroups. Differences between groups were considered significant when the p value was < 0.05.

## Supplementary Information

**Additional file 1.** ALKBH5 inhibited the proliferation of HLF and 97H cells, while knockdown of ALKBH5 in LM3 cells showed the opposite effect (A). Overexpression of ALKBH5 reduced the migration and invasion of HLF and 97H cells, while knockdown of ALKBH5 in LM3 cells showed the opposite effects (B–C).

**Additional file 2.** PAQR4 promoted the migration and invasion of HCC cells in vitro (A–B).

**Additional file 3.** Overexpression of ALKBH5 decreased the proliferation capacity in 2 HCC cell lines, while co-overexpressed of PAQR4 reverted this phenomenon (A and B). Overexpression of ALKBH5 decreased the invasion and migration ability, while co-overexpressed of PAQR4 reverted this phenomenon (C and D).

**Additional file 4.** Knockdown AKT significantly prevented the increases in proliferation, migration, and invasion induced by PAQR4 overexpression in HCC cells (A–C).

**Additional file 5: Table S1.** Correlation between PAQR4 and clinicopathological characteristics in HCC (n = 108).

## Data Availability

All data generated or analyzed during this study are included either in this article or in the additional files and methods, tables, figures, and figure legends files.

## Abbreviations

HCC: Hepatocellular carcinoma
ALKBH5: Alkb homolog 5
m6A: N6-methyladenosine
PAQR4: Progestin and AdipoQ Receptor 4
qRT-PCR: Quantitative reverse transcription PCR
RIP: RNA immunoprecipitation
MeRIP: Methylated RNA immunoprecipitation
EMT: Epithelial-to-mesenchymal transition
Den: Diethylnitrosamine

## References

[1] Siegel RL, Miller KD, Fuchs HE, Jemal A (2021). Cancer statistics. CA Cancer J Clin.

[2] Zhang J, He X, Wan Y, Zhang H, Tang T, Zhang M, Yu S, Zhao W, Chen L (2021). CD44 promotes hepatocellular carcinoma progression via upregulation of YAP. Exp Hematol Oncol.

[3] Moon AM, Singal AG, Tapper EB (2020). Contemporary epidemiology of chronic liver disease and cirrhosis. Clin Gastroenterol Hepatol.

[4] Yang S, Zhang H, Yang H, Zhang J, Wang J, Luo T, Jiang Y, Hua H (2021). SEPHS1 promotes SMAD2/3/4 expression and hepatocellular carcinoma cells invasion. Exp Hematol Oncol.

[5] Zhang SZ, Zhu XD, Feng LH, Li XL, Liu XF, Sun HC, Tang ZY (2021). PCSK9 promotes tumor growth by inhibiting tumor cell apoptosis in hepatocellular carcinoma. Exp Hematol Oncol.

[6] Zheng Y, Huang C, Lu L, Yu K, Zhao J, Chen M, Liu L, Sun Q, Lin Z, Zheng J (2021). STOML2 potentiates metastasis of hepatocellular carcinoma by promoting PINK1-mediated mitophagy and regulates sensitivity to lenvatinib. J Hematol Oncol.

[7] Ma JZ, Yang F, Zhou CC, Liu F, Yuan JH, Wang F, Wang TT, Xu QG, Zhou WP, Sun SH (2017). METTL14 suppresses the metastatic potential of hepatocellular carcinoma by modulating N 6 -methyladenosine-dependent primary MicroRNA processing. Hepatology.

[8] Aldrighetti L, Pulitano C, Catena M, Arru M, Guzzetti E, Halliday J, Ferla G (2009). Liver resection with portal vein thrombectomy for hepatocellular carcinoma with vascular invasion. Ann Surg Oncol.

[9] Jia G, Fu Y, Zhao X, Dai Q, Zheng G, Yang Y, Yi C, Lindahl T, Pan T, Yang Y-G (2011). N6-Methyladenosine in nuclear RNA is a major substrate of the obesity-associated FTO. Nat Chem Biol.

[10] Huang H, Weng H, Chen J (2020). m6A modification in coding and non-coding RNAs: roles and therapeutic implications in cancer. Cancer Cell.

[11] Shen S, Zhang R, Jiang Y, Li Y, Lin L, Liu Z, Zhao Y, Shen H, Hu Z, Wei Y (2021). Comprehensive analyses of m6A regulators and interactive coding and non-coding RNAs across 32 cancer types. Mol Cancer.

[12] Chen M, Wei L, Law CT, Tsang FH, Shen J, Cheng CL, Tsang LH, Ho DW, Chiu DK, Lee JM (2018). RNA N6-methyladenosine methyltransferase-like 3 promotes liver cancer progression through YTHDF2-dependent posttranscriptional silencing of SOCS2. Hepatology..

[13] Lin X, Chai G, Wu Y, Li J, Chen F, Liu J, Luo G, Tauler J, Du J, Lin S (2019). RNA m6A methylation regulates the epithelial mesenchymal transition of cancer cells and translation of Snail. Nat Commun.

[14] Du L, Li Y, Kang M, Feng M, Ren Y, Dai H, Wang Y, Wang Y, Tang B (2021). USP48 is upregulated by Mettl14 to attenuate hepatocellular carcinoma via regulating SIRT6 stabilization. Cancer Res.

[15] Huang Q, Mo J, Liao Z, Chen X, Zhang B (2022). The RNA m6A writer WTAP in diseases: structure, roles, and mechanisms. Cell Death Dis.

[16] Zheng G, Dahl JA, Niu Y, Fedorcsak P, Huang CM, Li CJ, Vagbo CB, Shi Y, Wang WL, Song SH (2013). ALKBH5 is a mammalian RNA demethylase that impacts RNA metabolism and mouse fertility. Mol Cell.

[17] Zhang S, Zhao BS, Zhou A, Lin K, Zheng S, Lu Z, Chen Y, Sulman EP, Xie K, Bögler O (2017). m 6 A demethylase ALKBH5 maintains tumorigenicity of glioblastoma stem-like Cells by sustaining FOXM1 expression and cell proliferation program. Cancer Cell.

[18] Qiu X, Yang S, Wang S, Wu J, Zheng B, Wang K, Shen S, Jeong S, Li Z, Zhu Y (2021). M6A demethylase ALKBH5 regulates PD-L1 expression and tumor immunoenvironment in intrahepatic cholangiocarcinoma. Can Res.

[19] Liu F, Qin L, Liao Z, Song J, Yuan C, Liu Y, Wang Y, Xu H, Zhang Q, Pei Y (2020). Microenvironment characterization and multi-omics signatures related to prognosis and immunotherapy response of hepatocellular carcinoma. Exp Hematol Oncol.

[20] Lu JC, Zhang PF, Huang XY, Guo XJ, Gao C, Zeng HY, Zheng YM, Wang SW, Cai JB, Sun QM (2021). Amplification of spatially isolated adenosine pathway by tumor-macrophage interaction induces anti-PD1 resistance in hepatocellular carcinoma. J Hematol Oncol.

[21] Nie S, Zhang L, Liu J, Wan Y, Jiang Y, Yang J, Sun R, Ma X, Sun G, Meng H (2021). ALKBH5-HOXA10 loop-mediated JAK2 m6A demethylation and cisplatin resistance in epithelial ovarian cancer. J Exp Clin Cancer Res.

[22] Chen Y, Zhao Y, Chen J, Peng C, Zhang Y, Tong R, Cheng Q, Yang B, Feng X, Lu Y (2020). ALKBH5 suppresses malignancy of hepatocellular carcinoma via m6A-guided epigenetic inhibition of LYPD1. Mol Cancer.

[23] Qu S, Jin L, Huang H, Lin J, Gao W, Zeng Z (2021). A positive-feedback loop between HBx and ALKBH5 promotes hepatocellular carcinogenesis. BMC Cancer.

[24] Zaccara S, Ries RJ, Jaffrey SR (2019). Reading, writing and erasing mRNA methylation. Nat Rev Mol Cell Biol.

[25] Huang X, Zhang H, Guo X, Zhu Z, Cai H, Kong X (2018). Insulin-like growth factor 2 mRNA-binding protein 1 (IGF2BP1) in cancer. J Hematol Oncol.

[26] Giannelli G, Koudelkova P, Dituri F, Mikulits W (2016). Role of epithelial to mesenchymal transition in hepatocellular carcinoma. J Hepatol.

[27] Zhao G, Shi X, Sun Z, Zhao P, Lu Z (2021). PAQR4 promotes the development of hepatocellular carcinoma by activating PI3K/AKT pathway. Acta Biochim Biophys Sin.

[28] Ye J, Gao M, Guo X, Zhang H, Jiang F (2020). Breviscapine suppresses the growth and metastasis of prostate cancer through regulating PAQR4-mediated PI3K/Akt pathway. Biomed Pharmacother.

[29] Yang W-L, Wang J, Chan C-H, Lee S-W, Campos AD, Lamothe B, Hur L, Grabiner BC, Lin X, Darnay BG (2009). The E3 ligase TRAF6 regulates Akt ubiquitination and activation. Science.

[30] Wang G, Long J, Gao Y, Zhang W, Han F, Xu C, Sun L, Yang SC, Lan J, Hou Z (2019). SETDB1-mediated methylation of Akt promotes its K63-linked ubiquitination and activation leading to tumorigenesis. Nat Cell Biol.

[31] Cui Q, Shi H, Ye P, Li L, Qu Q, Sun G, Sun G, Lu Z, Huang Y, Yang CG (2017). m(6)A RNA methylation regulates the self-renewal and tumorigenesis of glioblastoma stem cells. Cell Rep.

[32] Li F, Yi Y, Miao Y, Long W, Long T, Chen S, Cheng W, Zou C, Zheng Y, Wu X (2019). N6-methyladenosine modulates nonsense-mediated mRNA decay in human glioblastoma. Can Res.

[33] Li Z, Weng H, Su R, Weng X, Zuo Z, Li C, Huang H, Nachtergaele S, Dong L, Hu C (2017). FTO plays an oncogenic role in acute myeloid leukemia as a N(6)-methyladenosine RNA demethylase. Cancer Cell.

[34] Zhang Z, Liu F, Chen W, Liao Z, Zhang W, Zhang B, Liang H, Chu L, Zhang Z (2022). The importance of N6-methyladenosine modification in tumor immunity and immunotherapy. Exp Hematol Oncol.

[35] Yankova E, Blackaby W, Albertella M, Rak J, De Braekeleer E, Tsagkogeorga G, Pilka ES, Aspris D, Leggate D, Hendrick AG (2021). Small-molecule inhibition of METTL3 as a strategy against myeloid leukaemia. Nature.

[36] Huang J, Shao Y, Gu W (2021). Function and clinical significance of N6-methyladenosine in digestive system tumours. Exp Hematol Oncol.

[37] Zaccara S, Jaffrey SR (2020). A unified model for the function of YTHDF proteins in regulating m(6)A-modified mRNA. Cell.

[38] Wang W, Shao F, Yang X, Wang J, Zhu R, Yang Y, Zhao G, Guo D, Sun Y, Wang J (2021). METTL3 promotes tumour development by decreasing APC expression mediated by APC mRNA N6-methyladenosine-dependent YTHDF binding. Nat Commun.

[39] Huang H, Weng H, Sun W, Qin X, Shi H, Wu H, Zhao BS, Mesquita A, Liu C, Yuan CL (2018). Recognition of RNA N6-methyladenosine by IGF2BP proteins enhances mRNA stability and translation. Nat Cell Biol.

[40] Chen X, Zhou X, Wang X (2022). m(6)A binding protein YTHDF2 in cancer. Exp Hematol Oncol.

[41] Tang YT, Hu T, Arterburn M, Boyle B, Bright JM, Emtage PC, Funk WD (2005). PAQR proteins: a novel membrane receptor family defined by an ancient 7-transmembrane pass motif. J Mol Evol.

[42] Xu P, Jiang L, Yang Y, Wu M, Liu B, Shi Y, Shen Q, Jiang X, He Y, Cheng D (2020). PAQR4 promotes chemoresistance in non-small cell lung cancer through inhibiting Nrf2 protein degradation. Theranostics.

[43] Wang L, Zhang R, You X, Zhang H, Wei S, Cheng T, Cao Q, Wang Z, Chen Y (2017). The steady-state level of CDK4 protein is regulated by antagonistic actions between PAQR4 and SKP2 and involved in tumorigenesis. J Mol Cell Biol.

[44] Pedersen L, Panahandeh P, Siraji MI, Knappskog S, Lønning PE, Gordillo R, Scherer PE, Molven A, Teigen K, Halberg N (2020). Golgi-localized PAQR4 mediates antiapoptotic ceramidase activity in breast cancer. Can Res.

[45] Luo X, Cao M, Gao F, He X (2021). YTHDF1 promotes hepatocellular carcinoma progression via activating PI3K/AKT/mTOR signaling pathway and inducing epithelial-mesenchymal transition. Exp Hematol Oncol.

[46] Rebouissou S, Nault J-C (2020). Advances in molecular classification and precision oncology in hepatocellular carcinoma. J Hepatol.

[47] Song M, Bode AM, Dong Z, Lee M-H (2019). AKT as a therapeutic target for cancer. Can Res.

