# Supplementary figures and images for: ALKBH5 prevents hepatocellular carcinoma progression by post-transcriptional inhibition of PAQR4 in an m6A dependent manner
Source: Exp Hematol Oncol. 2023 Jan 6;12:1. doi: 10.1186/s40164-022-00370-2 (PMC9825045; doi:10.1186/s40164-022-00370-2)

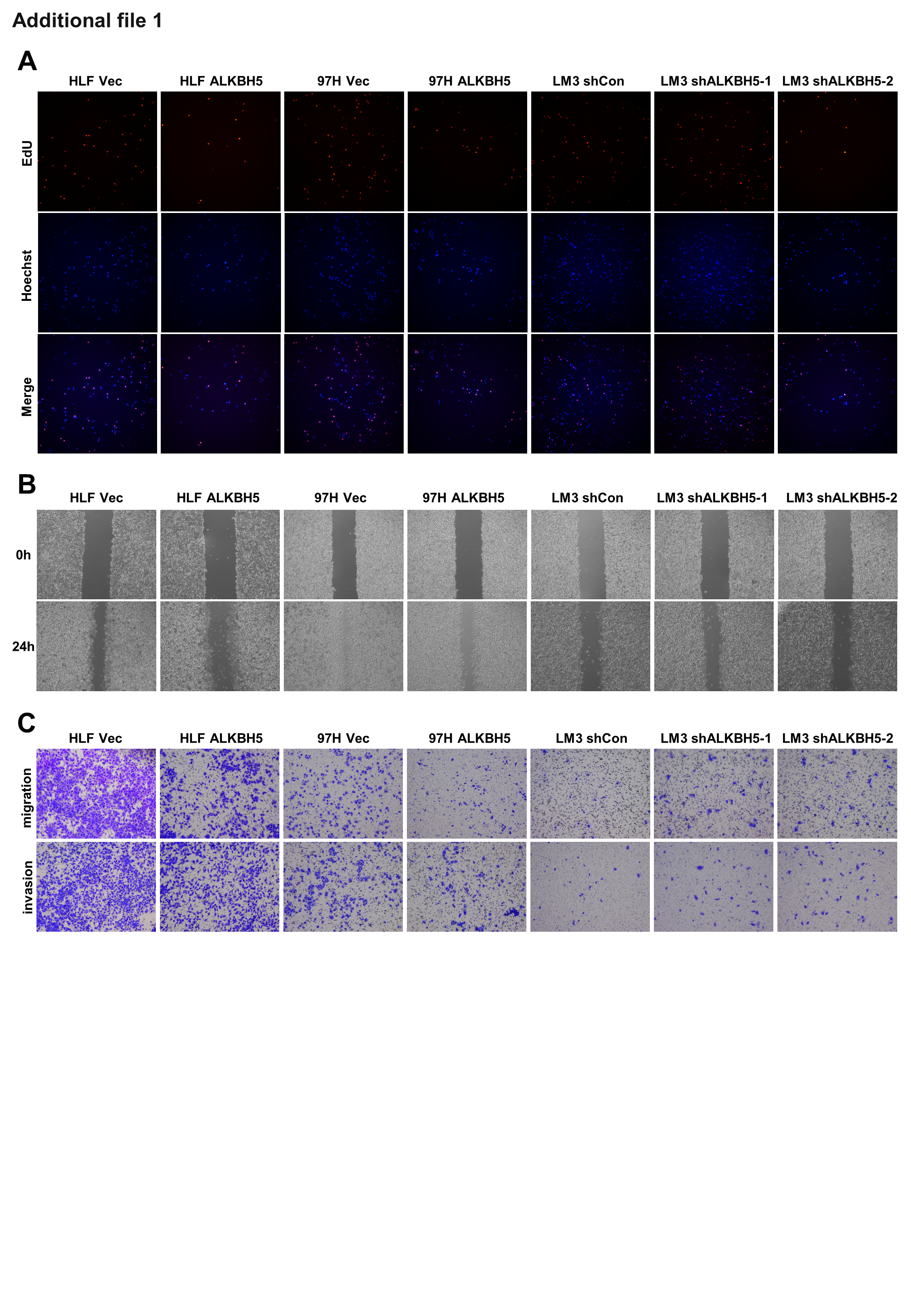

Supplement: Supplementary file 1 — Additional file 1. ALKBH5 inhibited the proliferation of HLF and 97H cells, while knockdown of ALKBH5 in LM3 cells showed the opposite effect (A). Overexpression of ALKBH5 reduced the migration and invasion of HLF and 97H cells, while knockdown of ALKBH5 in LM3 cells showed the opposite effects (B–C). [file 40164_2022_370_MOESM1_ESM.tif]

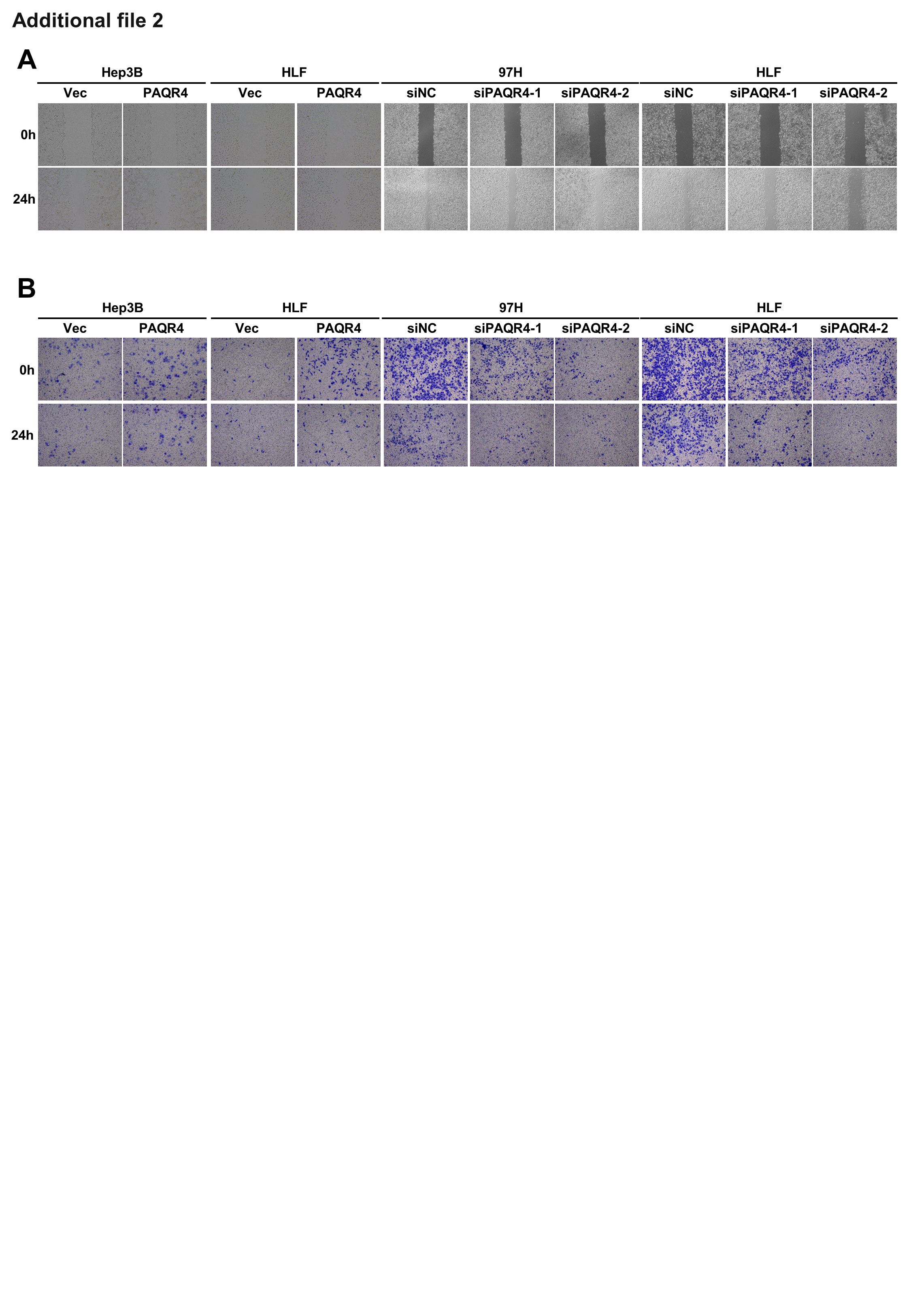

Supplement: Supplementary file 2 — Additional file 2. PAQR4 promoted the migration and invasion of HCC cells in vitro (A–B). [file 40164_2022_370_MOESM2_ESM.tif]

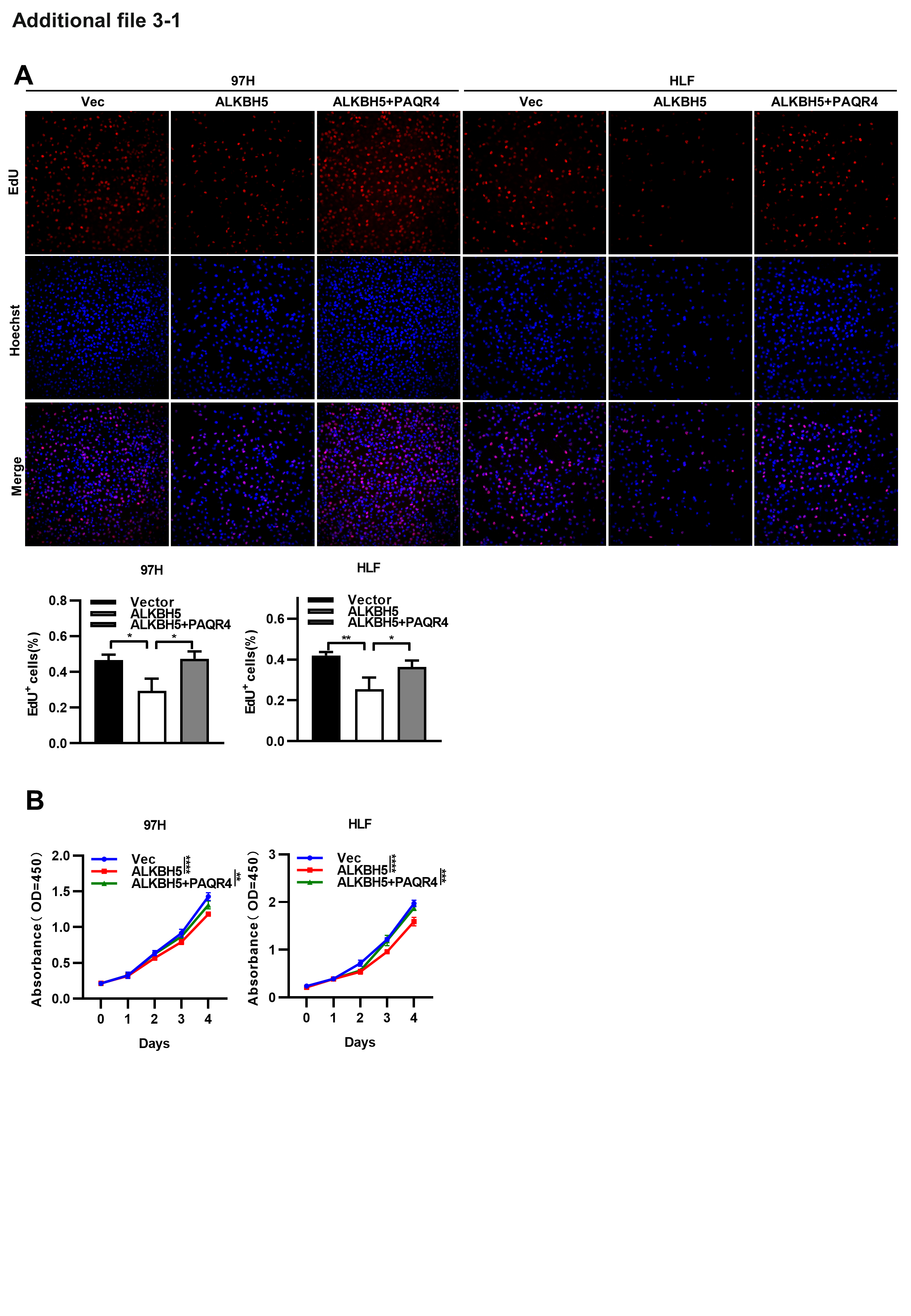

Supplement: Supplementary file 3 — Additional file 3. Overexpression of ALKBH5 decreased the proliferation capacity in 2 HCC cell lines, while co-overexpressed of PAQR4 reverted this phenomenon (A and B). Overexpression of ALKBH5 decreased the invasion and migration ability, while co-overexpressed of PAQR4 reverted this phenomenon (C and D). [file 40164_2022_370_MOESM3_ESM.zip › Additional file 3-1.TIF]

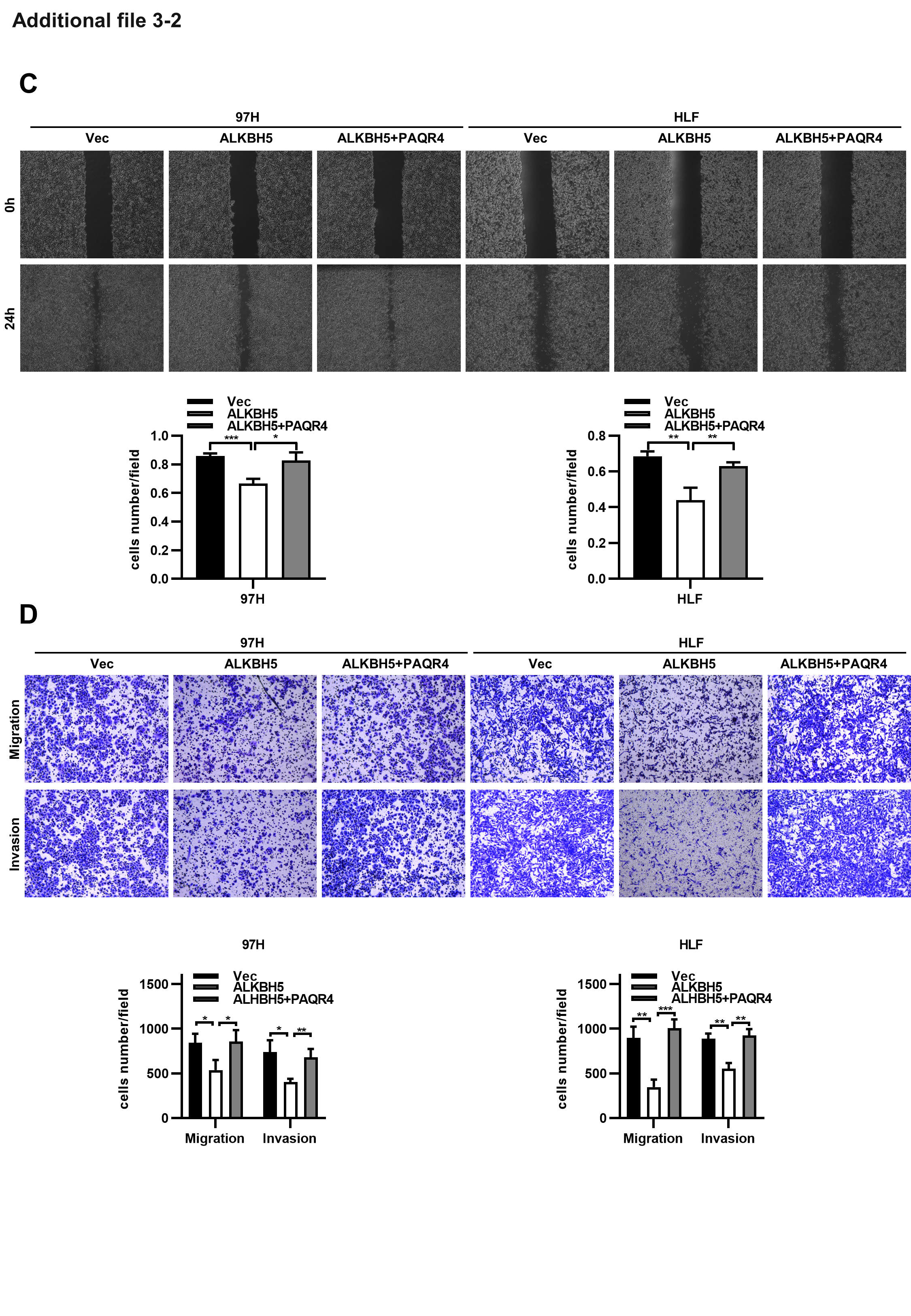

Supplement: Supplementary file 3 — Additional file 3. Overexpression of ALKBH5 decreased the proliferation capacity in 2 HCC cell lines, while co-overexpressed of PAQR4 reverted this phenomenon (A and B). Overexpression of ALKBH5 decreased the invasion and migration ability, while co-overexpressed of PAQR4 reverted this phenomenon (C and D). [file 40164_2022_370_MOESM3_ESM.zip › Additional file 3-2.TIF]

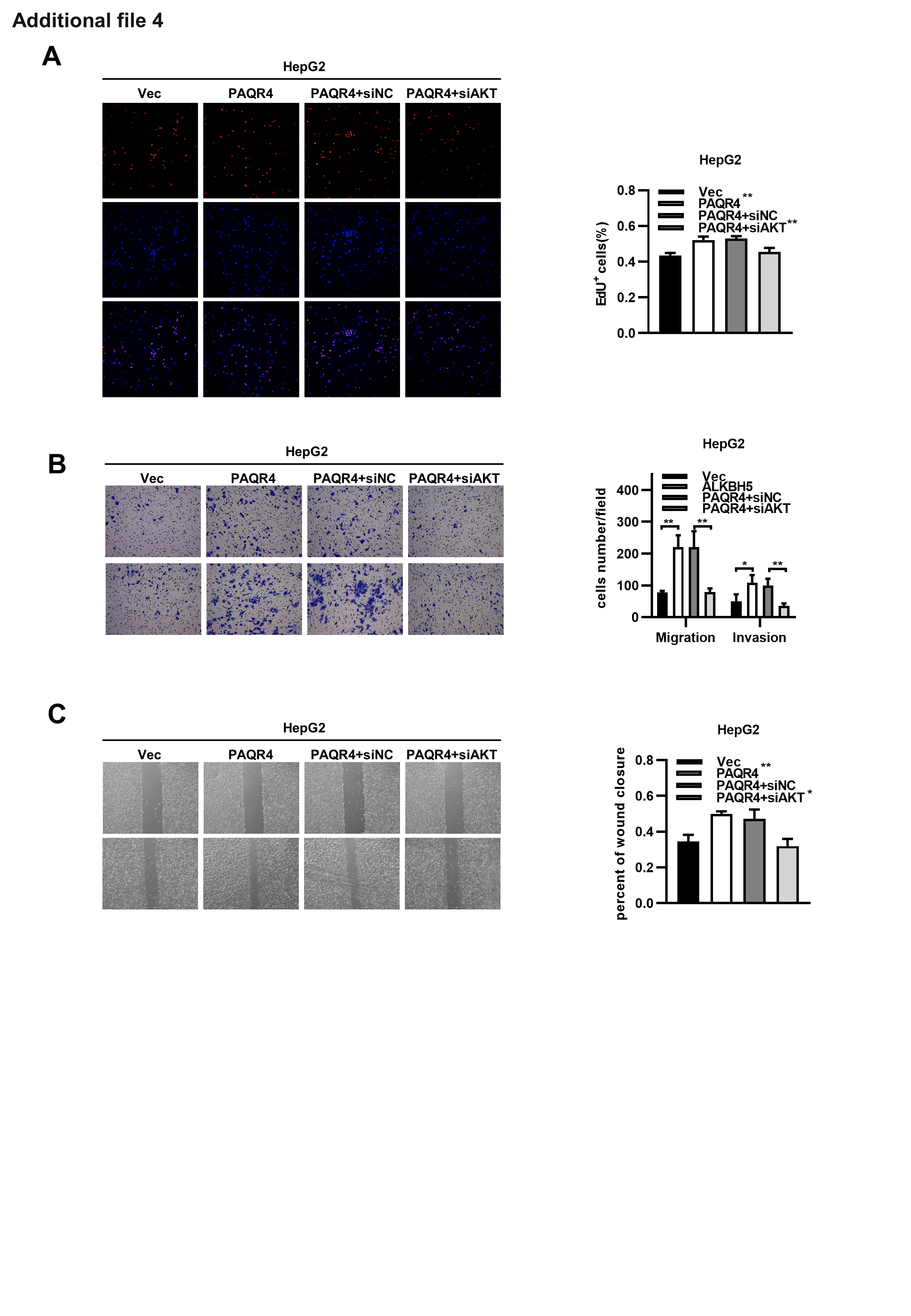

Supplement: Supplementary file 4 — Additional file 4. Knockdown AKT significantly prevented the increases in proliferation, migration, and invasion induced by PAQR4 overexpression in HCC cells (A–C). [file 40164_2022_370_MOESM4_ESM.tif]
