# Supplementary material for: ALKBH5 prevents hepatocellular carcinoma progression by post-transcriptional inhibition of PAQR4 in an m6A dependent manner
Source: Exp Hematol Oncol. 2023 Jan 6;12:1. doi: 10.1186/s40164-022-00370-2 (PMC9825045; doi:10.1186/s40164-022-00370-2)
Supplement: Supplementary file 5 — Additional file 5: Table S1. Correlation between PAQR4 and clinicopathological characteristics in HCC (n = 108). [file 40164_2022_370_MOESM5_ESM.docx]

**Table S1. Correlation between PAQR4 and clinicopathological characteristics in HCC (n=108).**

| **Clinicopathological**  **variables** | **Relative PAQR4 Expression** | | **P value** |
| --- | --- | --- | --- |
|  | **Low (52)** | **High (56)** |  |
| **Gender**  Male  Female | 42  10 | 46  10 | 0.854 |
| **Age**  ≤50  > 50 | 22  30 | 31  25 | 0.175 |
| **AFP (ug/L)**  ≤20  > 20 | 23  29 | 18  38 | 0.196 |
| **ALT(ng/ml)**  ≤75  >75 | 49  3 | 52  4 | 0.772 |
| **Cirrhosis**  No  Yes | 19  33 | 15  41 | 0.276 |
| **Tumor size (cm)**  ≤5  >5 | 18  34 | 21  35 | 0.755 |
| **Tumor encapsulation**  Complete  None | 29  23 | 20  36 | **0.036** |
| **Tumor number**  Single  Multiple | 40  12 | 38  18 | 0.067 |
| **Vascular invasion**  No  Yes | 36  16 | 37  19 | **0.038** |
| **Differentiation**  I- II  III-IV | 17  35 | 18  38 | 0.951 |
| **BCLC stage**  0+A  B+C | 38  14 | 29  27 | **0.023** |
